# Supplementary material for: BAF180 Promotes Cohesion and Prevents Genome Instability and Aneuploidy
Source: Cell Rep. 2014 Mar 6;6(6):973–81. doi: 10.1016/j.celrep.2014.02.012 (PMC3988838; doi:10.1016/j.celrep.2014.02.012)
Supplement: Document S1. Supplemental Experimental Procedures and Figures S1–S5 [file mmc1.pdf]

SUPPLEMENTAL INFORMATION

BAF180 promotes cohesion and prevents genome instability and aneuploidy

Brownlee, *et al.*

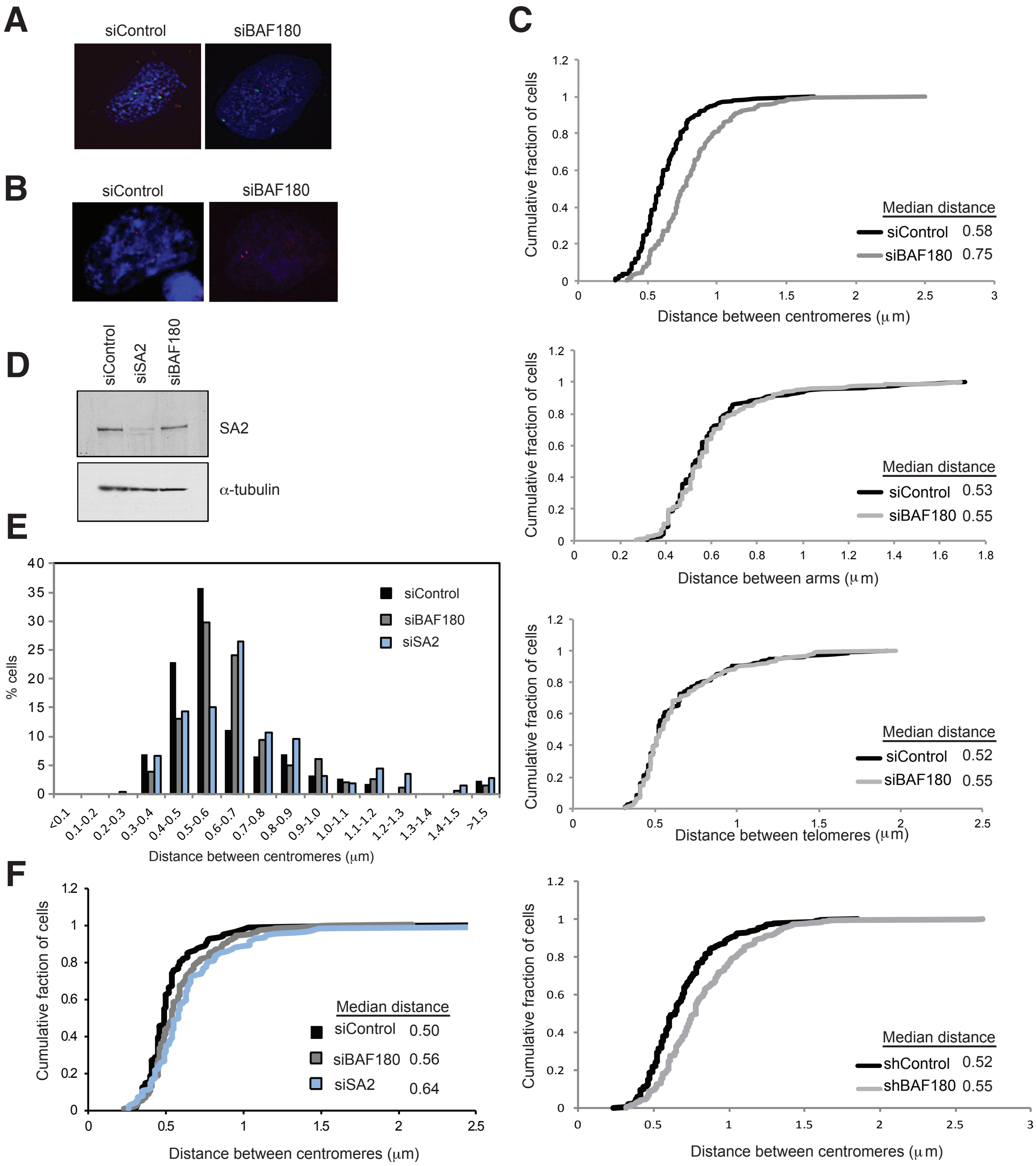

Fig. S1. Depletion of BAF180 results in defective cohesion at centromeres, Related to Figure 1. (A) Representative images of FISH analyses of si Control and siBAF180 1BR hTERT cells using a mixture of probes directed against the centromere (green) or arm (red) of chromosome 10. (B) Representative images of FISH analyses of siControl and siBAF180 1BR hTERT cells using a probe directed against the subtelomeric region of chromosome 16. (C) The data generated using these probes, presented in Figures 1D and F, are replotted here as cumulative plots and the median distances between sister chromatids is shown in each panel. Top three panels are of 1BR hTERT cells and the bottom panel is of the stable U2OS cell lines. (D) Western blot analysis of siRNA treated 1BR hTERT cells using antibodies directed against SA2 or tubulin. (E and F) siRNA treated cells as in D were analysed by FISH and distances between centromeres were measured and plotted as a distribution (E) or as a cumulative plot (F). Median distances are indicated in (F) and both BAF180- and SA2-depleted cells show a significant difference in distribution from control cells ($p=0.001$ and $p<0.001$, respectively).

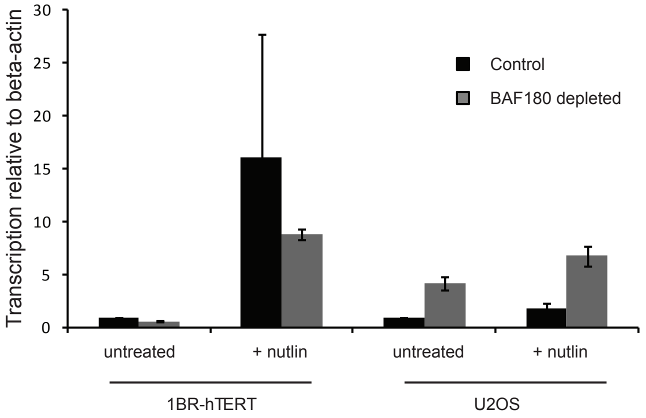

Fig S2. p21 transcription in 1BR-hTert and U20S cell lines in the absence and presence of nutlin, Related to Figure 2. Quantitative RT-PCR analysis of p21 transcripts in siControl and siBAF180 1BR hTERT cells (left) and shControl and shBAF180 U2OS cells (right) in the absence and presence of nutlin. Data are the average of three independent experiments and are expressed relative to a control transcript; beta-actin. For each cell line, the data have been normalized to the untreated control.

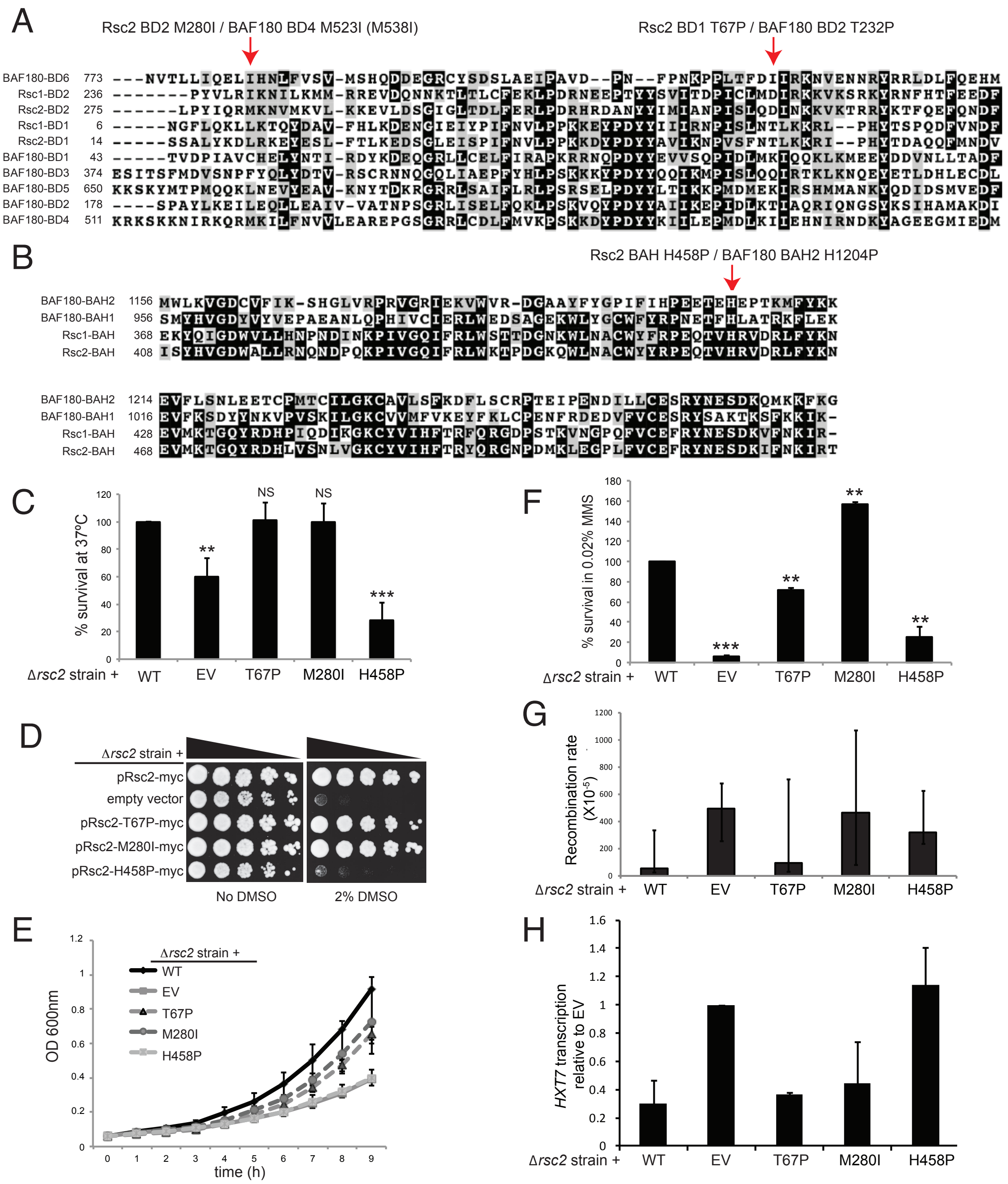

Fig. S3. Cancer associated mutations of BAF180 are conserved in the yeast homologues, and mutation impairs a subset of Rsc2 functions, Related to Figure 3. **(A)** Pilup of all six bromodomains of BAF180 with the bromodomains of Rsc1 and Rsc2. The cancer associated mutations of BAF180 are indicated (M523 corresponds to M538 of BAF180 isoform 8, used in our assays). **(B)** Pileup of the two BAH domains of BAF180 with the BAH domains from Rsc1 and Rsc2. The cancer associated H-P mutation is indicated. **(C)** Quantification of survival at 37ºC (ts phenotype) of wt and Rsc2 mutants. **(D)** Serial dilutions of mid-log cultures of the indicated strains were plated onto media with and without DMSO. **(E)** Growth curves of wt and mutant Rsc2-containing strains. **(F)** Quantification of survival in the presence of the DNA damaging agent methyl methane sulfonate (MMS) of wt and Rsc2 mutants. **(G)** Median rates of recombination with 10% and 90% confidence intervals (bars). **(H)** Quantitative RT-PCR analysis in *rsc2* cells harboring the indicated plasmid.

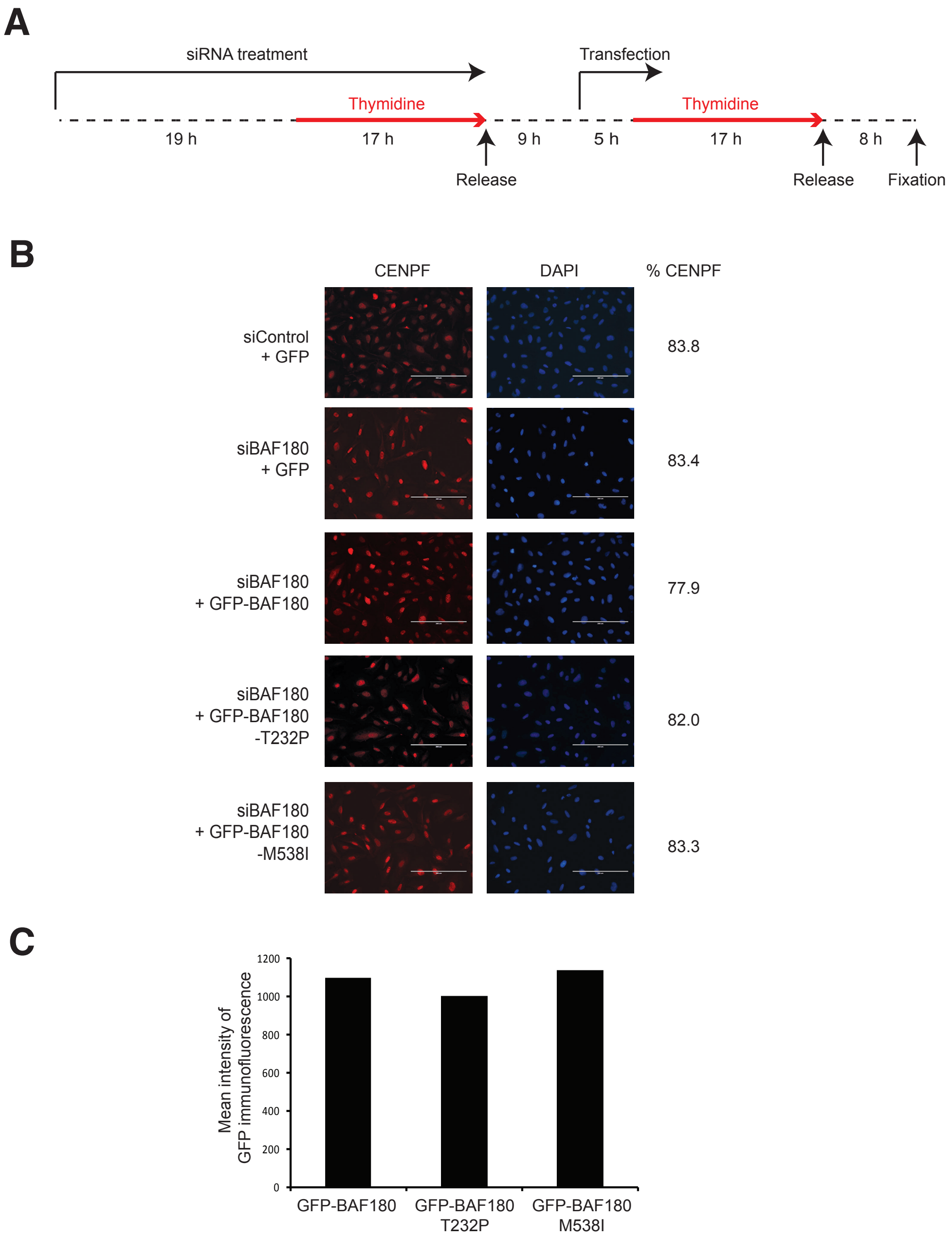

Fig. S4. Preparation of cells for cohesin analysis as in Figure 3. **(A)** Illustration of the protocol used to deplete BAF180, transfect with GFP constructs as in (B) and synchronize the cells in G2. **(B)** Representative images showing CENPF immunostaining, used as a marker of G2 cells. The percentage of CENPF positive cells is shown on the right. **(C)** Analysis of expression of GFP-BAF180 and mutants by immunofluorescence. Slides were prepared as in (A) and mean intensities were calculated from analysis of at least 60 cells.

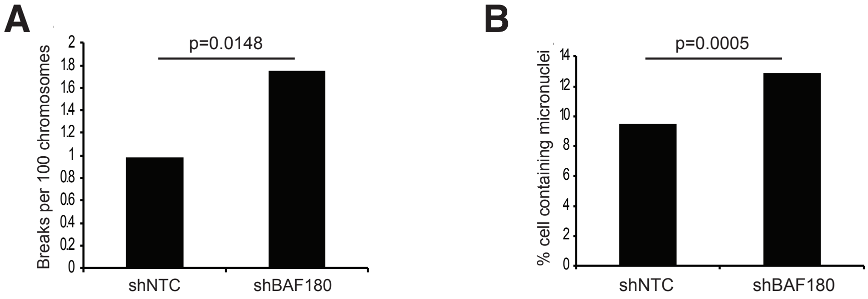

Fig. S5. Loss of BAF180 in U2OS cells leads to increased frequency of breaks and chromosomal instability on exposure to MMC, Related to Figure 5. **(A)** Analysis of chromosome and chromatid breaks in control or BAF180-depleted U2OS cells following exposure to 0.04 μg/ml MMC for 48 hours. A total of 2862 or 2741 chromosomes were analysed for control and BAF180-depleted cells, respectively, and data are expressed as breaks per 100 chromosomes. (B) Quantification of micronuclei in control or BAF180-depleted U2OS cells following exposure to MMC as in (A). A minimum of 1500 cells was scored for each cell line.

EXTENDED EXPERIMENTAL PROCEDURES

Cell culture

Mouse ES cells were cultured at 37°C in a 5% CO_2 incubator in Knockout™ DMEM (Gibco), supplemented with 15% ES-Cult FBS (Stemcell Technologies), 1% penicillin/streptomycin, 1% NEAA, 1% L-glutamine, 0.1 μM β-mercaptoethanol and 1000 U/ml LIF (Millipore). Cells were seeded into gelatin-coated dishes. 1BR-hTERT and U2OS cells were cultured at 37°C in a 5% CO_2 incubator in DMEM (Gibco) supplemented with 15% FBS, 1% penicillin/streptomycin and 1% L-glutamine.

siRNA knock-downs

1BR-hTERT and U2OS cells were plated at $1x10^5$ cells per 6cm dish and knock-downs performed as (Niimi et al., 2012). Cells were transfected with 20 nM BAF180 siRNA or 20 nM non-targeting control (ON-TARGET plus SMART pool, Dharmacon) using HiPerFect transfection reagent (Qiagen), and then again with the same siRNAs 24h later. Cells were treated or sampled 72h after the first transfection.

Antibodies and immunoblotting

Custom made and commercially available antibodies used in this study are as follows: Myc (9E10 clone, CRUK), BAF180 (A301-590A, Universal Biologicals Cambridge), Smc1 (A300-055A, Universal Biologicals Cambridge), Smc3 (ab155587, Abcam), SA1 (ab4457, Abcam), SA2 (ab4463, Abcam), β-actin (ab8226, Abcam), H3 (Abcam), α-tubulin (ab7750, Abcam), CENPF (ab108483, Abcam), GFP (A6455, Life Technologies), H2A (Downs et al., 2000). Samples for Western blot analysis were prepared from yeast by glass-bead disruption and TCA precipitation. For mammalian cell samples, cells were lysed by resuspension in Laemmli buffer.

Yeast strains and plasmids

RSC2 was deleted in DMY3010 (Huang et al., 2006) or BY471 backgrounds via standard gene-disruption methods using a cassette amplified by PCR. pRsc2-myc

(Chambers et al., 2013) was altered by site-directed mutagenesis to generate pRsc2-T67P-myc, pRsc2-M280I-myc, pRsc2-H458P-myc and pRsc2-D540G.

BAF180 expression plasmids

BAF180 complete cDNA (clone MGC:156155, IMAGE:40082629) was purchased from Source BioScience and cloned into the *Hind*III/*Kpn*I sites of pEGFP-C3 (Clontech) to generate pEGFP-BAF180. This clone contains a N122S substitution relative to the published sequence. To create the siRNA resistant construct pEGFP-BAF180r site-directed mutagenesis was performed using the following oligonucleotide sequence: GAGAAATCTTGAGACAGCCAAGAAA. Site-directed mutagenesis was performed on pEGFP-BAF180r to generate siRNA resistant plasmids expressing EGFP-tagged BAF180 containing substitutions identified in tumour samples: pEGFP-BAF180r-T232P and pEGFP-BAF180r-M538I.

Analysis of yeast transcription

Δ*rsc2* DMY3010 containing pRS415 (empty vector), pRsc2-myc (wt), pRsc2-T67P-myc, pRsc2-M280I-myc or pRsc2-H458P-myc were grown to mid-log phase in media lacking tryptophan and RNA extracted using an RNeasy kit (Qiagen). 1 μg of RNA was reverse transcribed into cDNA for analysis by qPCR using primers specific to the *HXT7* locus.

SUPPLEMENTAL REFERENCES

Chambers, A.L., Pearl, L.H., Oliver, A.W., and Downs, J.A. (2013). The BAH domain of Rsc2 is a histone H3 binding domain. Nucleic Acids Res *41*, 9168-9182.

Downs, J.A., Lowndes, N.F., and Jackson, S.P. (2000). A role for Saccharomyces cerevisiae histone H2A in DNA repair. Nature *408*, 1001-1004.

Huang, J., Brito, I.L., Villen, J., Gygi, S.P., Amon, A., and Moazed, D. (2006). Inhibition of homologous recombination by a cohesin-associated clamp complex recruited to the rDNA recombination enhancer. Genes Dev *20*, 2887-2901.

Niimi, A., Chambers, A.L., Downs, J.A., and Lehmann, A.R. (2012). A role for chromatin remodellers in replication of damaged DNA. Nucleic acids research.
