# Supplementary material for: BAF180 Promotes Cohesion and Prevents Genome Instability and Aneuploidy
Source: Cell Rep. 2014 Mar 6;6(6):973–81. doi: 10.1016/j.celrep.2014.02.012 (PMC3988838; doi:10.1016/j.celrep.2014.02.012)
Supplement: Document S2. Article plus Supplemental Information [file mmc2.pdf]

Cell Reports
Report

BAF180 Promotes Cohesion and Prevents Genome Instability and Aneuploidy

Peter M. Brownlee,[1] Anna L. Chambers,[1] Ross Cloney,[1] Alessandro Bianchi,[1] and Jessica A. Downs[1,*]
[1]MRC Genome Damage and Stability Centre, University of Sussex, Falmer, Brighton BN1 9RQ, UK
*Correspondence: j.a.downs@sussex.ac.uk
http://dx.doi.org/10.1016/j.celrep.2014.02.012
This is an open access article under the CC BY license (http://creativecommons.org/licenses/by/3.0/).

SUMMARY

BAF180, a subunit of the PBAF chromatin remodeling complex, is frequently mutated in cancer. Although PBAF regulates transcription, it remains unclear whether this is what drives tumorigenesis in cells lacking BAF180. Based on data from yeast, we hypothesized that BAF180 may prevent tumorigenesis by promoting cohesion. Here, we show BAF180 is required for centromeric cohesion in mouse and human cells. Mutations identified in tumor samples are unable to support this activity, and also compromise cohesion-dependent functions in yeast. We provide evidence of genome instability in line with loss of cohesion, and importantly, we find dynamic chromosome instability following DNA damage in cells lacking BAF180. These data demonstrate a function for BAF180 in promoting genome stability that is distinct from its well-characterized role in transcriptional regulation, uncovering a potent mechanism for its tumor-suppressor activity.

INTRODUCTION

PBAF is a SWI/SNF chromatin remodeling complex found in mammalian cells. BAF180 is one of three subunits that distinguish PBAF (or SWI/SNF-B) from the other SWI/SNF complex, termed BAF (or SWI/SNF-A). Recent exome sequencing studies led to the unexpected finding that SWI/SNF subunits are mutated at a high frequency in many different cancer types, and in particular, mutations in *PBRM1*, which encodes BAF180, were frequently identified, including in over 40% of renal cell carcinoma samples (Shain and Pollack, 2013; Varela et al., 2011; Xia et al., 2008), indicating that BAF180 plays a critical role in preventing tumorigenesis. Even though BAF180 was identified as a regulator of p53-dependent transcriptional activity (Burrows et al., 2010; Xia et al., 2008), it is unclear whether this is the mechanism by which it functions as a tumor-suppressor gene.

BAF180 is a large protein with multiple domains, including six bromodomains (BDs) and two bromo-adjacent homology (BAH) domains. In yeast, these domains are encoded by three separate subunits, Rsc1, Rsc2, and Rsc4, which are part of the RSC chromatin remodeling complex. In addition to regulating gene transcription, RSC is important for sister chromatid cohesion (Baetz et al., 2004; Huang et al., 2004). Notably, recent reports demonstrated that defective cohesion results in chromosomal instability (CIN) and aneuploidy, and leads to tumorigenesis in mammalian cells (Carretero et al., 2013; Guo et al., 2013; Remeseiro et al., 2012; Solomon et al., 2011, 2013). Moreover, aneuploidy itself can drive further genome instability and can also, in certain contexts, contribute to tumorigenesis (for review, see Holland and Cleveland, 2012). These observations raise the possibility that a major mechanism by which BAF180 functions as a tumor suppressor is by promoting sister chromatid cohesion.

RESULTS

BAF180 Contributes to Sister Chromatid Cohesion Specifically at Centromeres in Mammalian Cells

To test whether BAF180 is important for sister chromatid cohesion, we first prepared chromosome spreads from wild-type (WT) or BAF180 knockout mouse embryonic stem cells (mESCs) (Wang et al., 2004). When we scored for cohesion at the centromere, we found that the BAF180$^{-/-}$ cells showed a significant increase in the proportion of cells that displayed aberrant cohesion compared with the WT control cells (Figure 1A).

In higher eukaryotes, the cohesin complex can contain either SA1 (STAG1) or SA2 (STAG2), and recently it was found that SA1 is required for cohesion between sister telomeres, whereas SA2 is required for centromeric cohesion (Canudas and Smith, 2009; Remeseiro et al., 2012). We therefore scored cohesion at the chromosome arms to determine whether there was also a defect, and found no statistically significant difference between the BAF180$^{+/+}$ and BAF180$^{-/-}$ mESCs (Figure 1B), suggesting that BAF180 specifically promotes cohesion at the centromeres.

To further examine the cohesion defect, we depleted BAF180 using small interfering RNA (siRNA) in human fibroblast cells (1BR hTERT; Figure 1C) and performed fluorescence in situ hybridization (FISH; Figure S1). We first measured the distance between sister chromatids in mitotic cells using a probe specific to centromere 10. Compared with cells transfected with a nontargeting control construct, we found a shift in the distribution of distances between sister chromatids at the centromere (Figures 1D, top panel, and S1), consistent with the analysis of cohesion in mESCs.

Cell PRESS
Open ACCESS

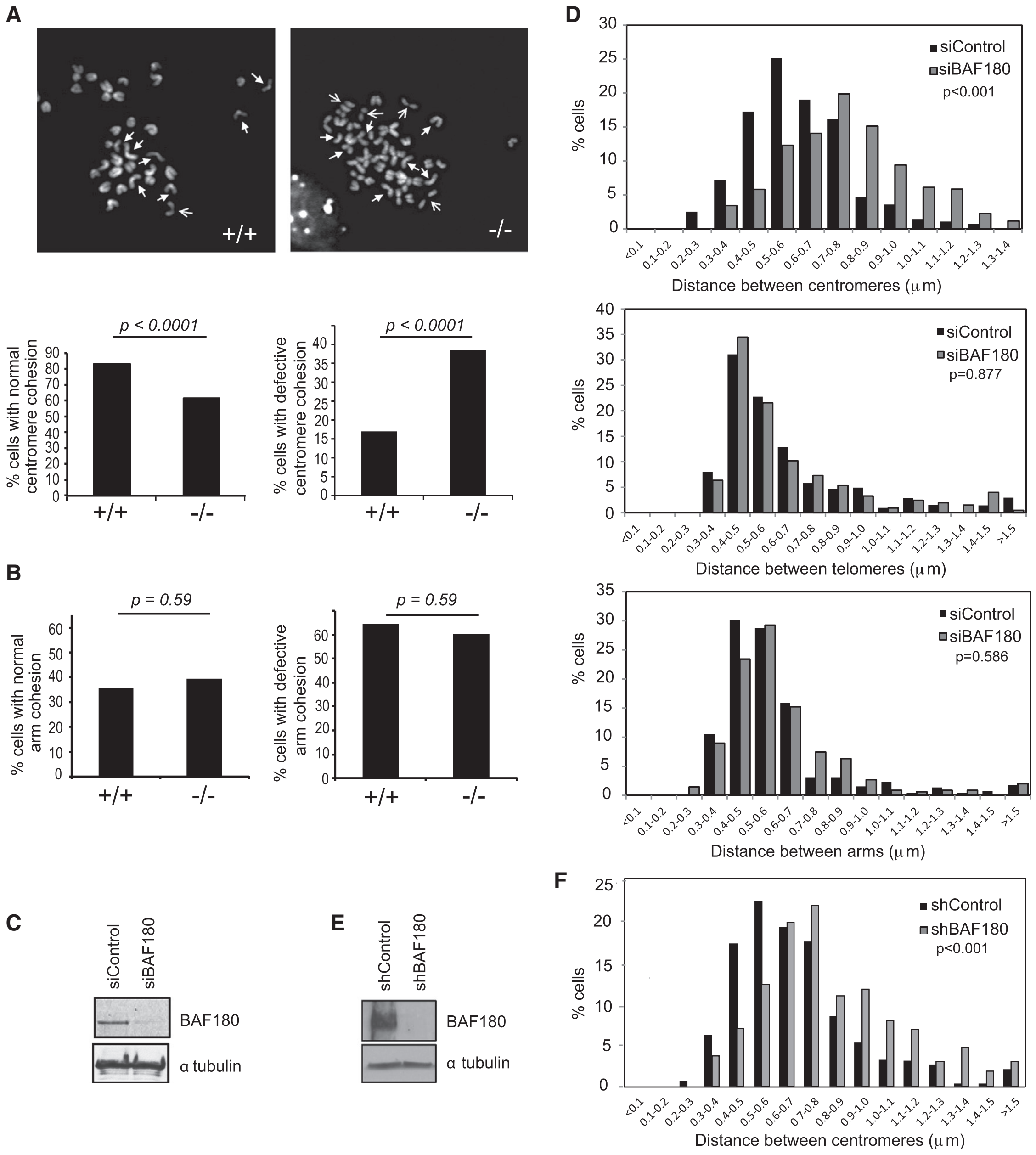

Figure 1. BAF180 Promotes Centromeric Sister Chromatid Cohesion

(A and B) Mitotic spreads prepared from WT (+/+) and BAF180 knockout (−/−) mESCs were analyzed for sister chromatid cohesion at either centromeres (A) or arms (B). Representative images from WT and BAF180$^{-/-}$ mESCs are shown in the top panels. Cells were analyzed according to whether cohesion was defective at centromeres (open arrows) or arms (open and closed arrows), and cells were scored as "normal" when two or fewer chromosomes showed defects, or "defective" when three or more chromosomes showed defects; 200 cells were scored per genotype.

(C) Analysis of BAF180 depletion efficiency in 1BR-hTERT cells by western blotting. Anti-tubulin was used as a loading control.

(D) FISH analysis of siControl and siBAF180 1BR-hTERT cells using probes directed against the centromere (top, $p < 0.001$), telomere (middle, $p = 0.877$), or chromosome arm (lower panel, $p = 0.586$). The distances between signals were measured from two independent experiments and the distribution was plotted as a histogram.

(E) Analysis of BAF180 protein levels in U2OS shBAF180 and shControl stable cells by western blotting. Anti-tubulin was used as a loading control.

(F) FISH analysis of shControl and shBAF180 U20S cells using a probe directed against the centromere ($p < 0.001$). The distances between signals were measured from two independent experiments and the distribution was plotted as a histogram.

See also Figure S1.

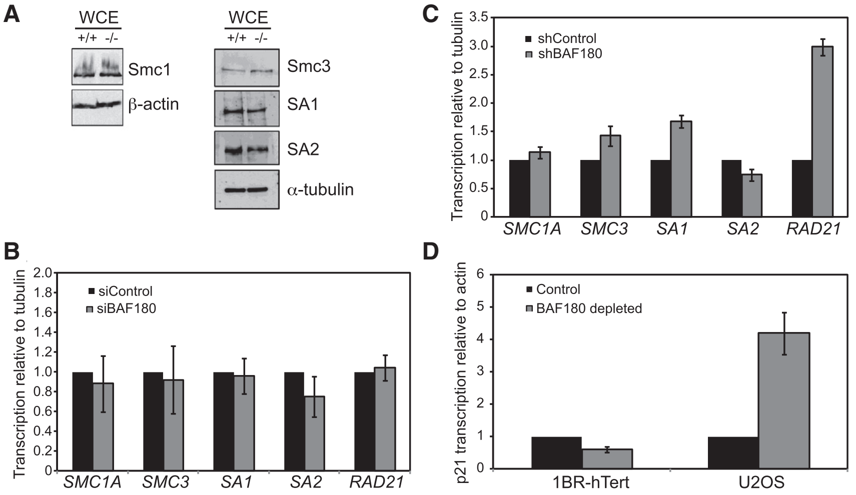

Figure 2. BAF180 Is Not Required for Transcription of Cohesin Genes and Has Tissue-Specific Roles in Regulating p53-Dependent p21 Transcription

(A) Western blot analysis of cohesin subunits in WCE prepared from WT (+/+) and BAF180 knockout (−/−) mESCs.

(B) Transcription of cohesin subunits in BAF180-depleted 1BR-hTert cells. Transcript levels from three independent experiments were analyzed by qPCR and normalized to α-tubulin transcript. Data for BAF180-depleted cells are shown relative to siControl cells.

(C) Transcription of cohesin subunits in shBAF180 U2OS cells. Transcript levels from three independent experiments were analyzed by qPCR and normalized to α-tubulin. Data for shBAF180 cells are shown relative to shControl cells.

(D) Transcription of p21 in BAF180-depleted 1BR-hTert and U2OS cells. Transcript levels from three independent experiments were analyzed by qPCR and normalized to β-actin. Data for BAF180-depleted cells are shown relative to control cells.

See also Figure S2.

The data we obtained from mouse cells indicated that the defect in cohesion is specific to the centromere; therefore, we tested whether BAF180 is also important for mediating cohesion between telomeres. Using a probe against the subtelomeric region of chromosome 16, we measured the distribution of distances between sister chromatids in siBAF180 and siControl cells as above. In contrast to our results with the centromeric probe, we found no significant difference in the BAF180-depleted cells compared with the control cells (Figures 1D, middle panel, and S1). Finally, we used a probe against the chromosome arm (20p12), and found no substantial difference between siBAF180 and control cells (Figures 1D, bottom panel, and S1). These data are similar to results obtained with SA2-depleted cells (Canudas and Smith, 2009), so we performed knockdown of SA2 and repeated the analysis of centromeres by FISH in order to compare the defect with that of BAF180. We found that the shift in distribution of distances between centromeres was slightly greater than that of BAF180 (Figure S1), suggesting that loss of BAF180 may not be as deleterious as loss of SA2.

To consolidate these data, and for use in future experiments, we also created a BAF180 small hairpin RNA (shRNA) stable cell line (and shControl cell line) in U2OS cells (Figure 1E). We analyzed these cells by FISH as described above using the centromere-specific probe, and found that, consistent with the data from mESCs and human fibroblast cells, U2OS cells depleted of BAF180 have a defect in cohesion at the centromere (Figure 1F). Together, these data suggest that BAF180 plays a conserved role in mediating centromeric sister chromatid cohesion in mammalian cells.

BAF180 Is Not Required for Transcription of Cohesin Genes and Has Tissue-Specific Roles in Regulating p53-Dependent p21 Transcription

One possible mechanism by which BAF180 mediates sister chromatid cohesion in cells is transcriptional regulation of cohesin genes. In argument against this, a microarray analysis of BAF180-depleted renal cell carcinomas did not show significant misregulation of cohesin genes (Varela et al., 2011). Moreover, we saw no gross differences in the protein levels of core cohesin subunits when whole-cell extracts from BAF180$^{+/+}$ and BAF180$^{-/-}$ cells were analyzed by western blotting (Figure 2A).

Nevertheless, to look at this directly in the human cell lines used in these assays, we examined the mRNA levels of the core cohesin genes *SMC1A*, *SMC3*, *SA1* (*STAG1*), *SA2* (*STAG2*), and *RAD21* in siBAF180-treated 1BR hTERT cells and the stable shBAF180 U2OS cells, and compared them with controls using quantitative RT-PCR (qRT-PCR). BAF180 depletion did not result in significantly decreased levels of any of these transcripts (Figures 2B and 2C). In fact, in the shBAF180 U2OS cells, the *RAD21* transcript, and to a lesser extent the *SMC3* and *SA1* transcripts, appeared to be upregulated. Together, these data suggest that the defect in cohesion is unlikely to be due to indirect transcriptional effects.

BAF180 has been implicated in regulating transcription of the p53-dependent p21 gene (Burrows et al., 2010; Xia et al., 2008), and this is certainly a mechanism by which loss of BAF180 may promote tumorigenesis. We set out to investigate the transcriptional status of p21 in our BAF180-depleted cell lines and found that, consistent with previous reports, the basal levels of p21 transcription were defective in a human fibroblast cell line (Figure 2D). Moreover, we examined induced p21 transcription by treating cells with the MDM2 inhibitor nutlin, and found that these transcripts were also downregulated when BAF180 was depleted in the 1BR hTERT cells (Figure S2).

In contrast, however, both basal and induced p21 transcripts were upregulated in the U2OS shBAF180 cells relative to the shControl cells (Figures 2D and S2), suggesting that p53-dependent transcriptional activation of p21 is BAF180 independent in these cells. This finding fortuitously allowed us to examine the effects of BAF180-dependent effects on cohesion in a cell line where p21 transcription is not reduced.

Cell PRESS
Open ACCESS

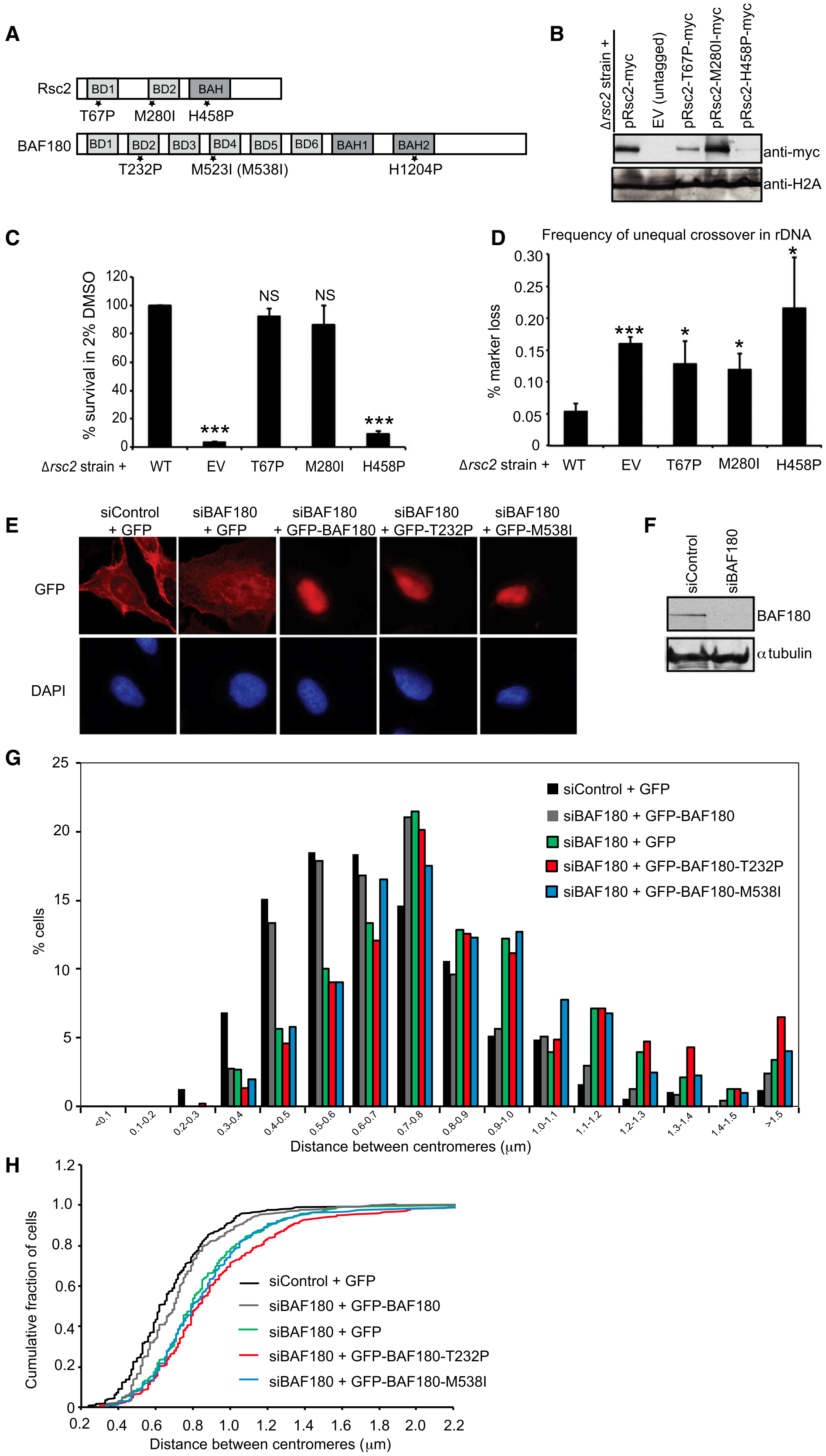

(legend on next page)

Cancer-Associated Mutations of BAF180 Impair Cohesion in Mammalian Cells and Cohesin-Dependent Functions in Budding Yeast

A number of missense mutations were identified in the gene encoding BAF180 (*PBRM1*) in cancer cells (Varela et al., 2011), some of which are predicted to have relatively little effect on protein folding and stability (Brownlee et al., 2012). We considered the possibility that these mutations might provide some insight into the mechanism(s) by which BAF180 suppresses tumorigenesis. We began by looking at the effects of these cancer mutations in the yeast homolog of BAF180.

We selected three mutations of BAF180 identified in cancer cells: T232P, M523I, and H1204P. The first two reside in BD2 and BD4 of BAF180, and the last mutation is found within the second BAH domain of BAF180. When aligned with Rsc1 and Rsc2, all three residues are conserved within Rsc2 (Figures 3A, S3A, and S3B; corresponding to T67 in BD1, M280 in BD2, and H458P in the BAH domain, respectively). Although two of the residues are also conserved within Rsc1 (Figures S3A and S3B), Rsc2 has a greater effect on DNA damage responses and cohesion (Baetz et al., 2004; Chambers et al., 2012), so we introduced the mutations into Rsc2.

We first transformed WT and mutant Rsc2 expression constructs into *rsc2* null yeast and then performed western blot analyses of whole-cell extracts to determine whether they had any effect on protein stability. We detected a very low level of Rsc2-H458P mutant protein compared with the WT control, suggesting that this mutation severely impairs protein stability. The Rsc2-T67P mutant protein was detected at intermediate levels, consistent with a slight destabilizing effect, and the Rsc2-M280I was similar to WT (Figure 3B).

Cells lacking *RSC2* have a growth defect and are temperature sensitive. They are also hypersensitive to DMSO, which likely reflects transcriptional misregulation of genes involved in cell wall biosynthesis (Angus-Hill et al., 2001). We found the Rsc2-H458P mutant strain showed phenotypes similar to those of the null strain (Figures 3C and S3), consistent with the greatly reduced protein levels. In contrast, both of the other mutant proteins were able to rescue the temperature-sensitivity (ts) and DMSO-hypersensitivity phenotypes of the *rsc2* null strain to apparently WT levels (Figures 3C and S3), as well as to restore the WT levels of the *HXT7* transcript, which is Rsc2 dependent (Figure S3). These results indicate that the Rsc2-T67P and Rsc2-M280I mutant proteins are still at least partly functional in vivo. In contrast, we found that all of the cancer-mutant-bearing strains showed statistically significant differences in survival relative to WT following DNA damage, and none of the cancer-mutant constructs were able to fully complement the growth defect (Figure S3).

The yeast phenotypes described above could potentially reflect a loss of cohesion-dependent activities in the cancer-mutant-containing strains. We therefore tested the mutant Rsc2 proteins using a recombination assay in which a reporter construct was integrated into the rDNA repeats. Strains with defective cohesion showed elevated rates of marker loss compared with WT, indicative of increased unequal sister chromatid exchange events (Huang and Moazed, 2003). As expected, the *rsc2* null cells showed a 3-fold increase in marker loss compared with the WT control (Figure 3D). Notably, we found that none of the cancer mutations were able to fully complement this activity (Figure 3D). These data are consistent with the idea that some cancer-associated mutations do not compromise all functions of Rsc2, but do compromise the cohesion-related functions of *RSC2* and result in genome instability.

We next created siRNA-resistant GFP-tagged WT and mutant expression constructs of BAF180. We focused on the two mutants that were expressed at reasonable levels in yeast: Rsc2-T67P and M280I. These correspond to BAF180 T232 and M538 in our construct (isoform 8; Figure S3). We then transfected these constructs into BAF180-depleted cells alongside WT and empty vector, synchronized them in G2, and analyzed them by immunofluorescence (IF)-FISH to examine the distance between sister chromatids in the transfected cells (Figures 3E, 3F, and S4). We found that expression of the mutants was comparable to that of the WT construct (Figures 3E and S4), but although the WT construct was able to restore cohesion, neither of the cancer-associated mutant constructs could (Figures 3G and 3H), suggesting that tumor cells bearing these mutations have compromised centromeric cohesion.

Loss of BAF180 Leads to Dynamic CIN

Defective cohesion leads to both structural and numerical CIN, due in part to problems with chromosome segregation leading

Figure 3. Mutations Identified in BAF180 from Cancer Samples Result in Impaired Cohesin-Dependent Functions in Yeast and Mammals

(A) Illustration of domain organization and relative position of cancer-associated mutations in Rsc2 and BAF180. BDs and BAH domains are numbered sequentially.

(B) Analysis of WT and mutant Rsc2 expression levels in total protein preparations by western blotting. Loading control: anti-H2A.

(C) Hypersensitivity to DMSO as a readout of Rsc2-dependent transcriptional activity was analyzed by plating serial dilutions of the indicated mid-log cultures onto media with or without 2% DMSO.

(D) Frequency of unequal rDNA crossover events in yeast strains containing the indicated Rsc2 expression construct.

(E) Expression of GFP-tagged BAF180 constructs in siBAF180 U20S. Cells were treated as in (G) and Figure S4, for IF-FISH. IF using anti-GFP shows GFP or GFP-BAF180 expression in the red channel.

(F) Analysis of BAF180 depletion efficiency in U2OS cells by western blotting. Anti-tubulin was used as a loading control.

(G) FISH analysis of G2 phase U2OS cells transfected with the indicated BAF180 expression construct using a probe against centromere 10. The distances between signals were measured and the distribution was plotted as a histogram.

(H) The data in (G) are presented as a cumulative plot to further illustrate the defect in cohesion in cells transfected with cancer-associated mutants. Statistical analysis of the data presented in (G) and (H) showed that rescue of the cohesion defect by reintroduction of WT BAF180 (siBAF180 + GFP-BAF180) was significant ($p < 0.001$). In contrast, centromeric cohesion in cells expressing the cancer mutants was not significantly different from that in BAF180-depleted cells containing empty vector (siBAF180 + GFP; $p = 0.06$ for T232P and $p = 0.37$ for M538I), but was significantly different from that in cells with WT BAF180 reintroduced ($p < 0.001$ for both mutants compared with WT).

See also Figures S3 and S4.

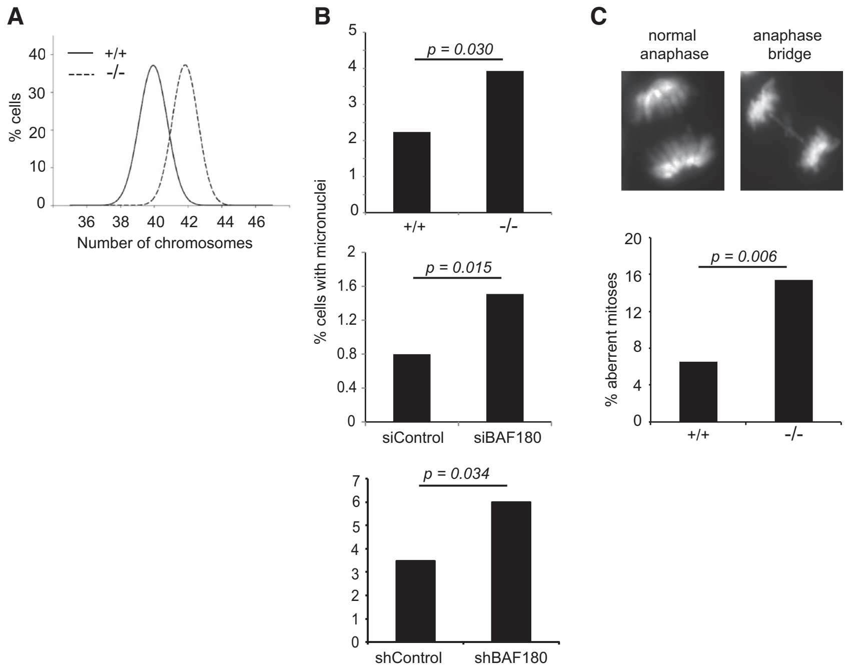

Figure 4. Cells Lacking BAF180 Are Aneuploid and Show Evidence of CIN

(A) Distribution curves showing chromosome numbers from WT (+/+) and BAF180 knockout (−/−) mESCs. Chromosomes were counted in 100 cells for each cell line using DAPI-stained metaphase spreads.

(B) Quantification of micronuclei present in WT (+/+) or BAF180 knockout (−/−) mESCs (top panel), control and BAF180-depleted 1BR-hTERT cells (middle panel), and shControl and shBAF180 U2OS cells (bottom panel). A minimum of 900 cells were scored for each cell line, and siRNA-depleted cells from three independent experiments were analyzed.

(C) Incidence of abnormal events during anaphase were quantified in WT (+/+; n = 183) and BAF180 knockout (−/−; n = 207) mESCs.

to aneuploidy, and defective recombination-based repair leading to structural chromosome aberrations (Xu et al., 2011). Cells lacking SA2, which have centromeric cohesion defects similar to BAF180-dependent effects, are aneuploid and show evidence of both structural and numerical CIN (Solomon et al., 2011). However, the effect of BAF180 depletion on sister chromatid cohesion is not as pronounced as that reported for SA2-depleted cells (Figure S2; Canudas and Smith, 2009), raising the possibility that this defect is not sufficient to disrupt chromosomal stability and therefore may not impact tumorigenesis. We therefore investigated whether loss of BAF180 leads to CIN.

We first determined whether BAF180 influences aneuploidy by analyzing chromosome spreads prepared from BAF180$^{-/-}$ mESCs, and found that they had an increased average number of chromosomes per cell when compared with WT (Figure 4A). Next, we investigated other readouts of CIN. Micronuclei can arise as a consequence of chromosome missegregation and are commonly seen in cells with defective cohesion (Barber et al., 2008; Musio et al., 2003). We observed an increase in the number of micronuclei present in the BAF180$^{-/-}$ mESCs compared with WT (Figure 4B, top panel). A similar increase was apparent when we examined the human cell lines depleted of BAF180 (siBAF180 1BR hTERT cells and shBAF180 U2OS) when compared with their controls (Figure 4B, middle and lower panels). Notably, the result obtained in the siBAF180-treated cells suggests that the effect of BAF180 loss on genome stability is rapid and does not require extensive cell passaging. Further, imaging of BAF180$^{-/-}$ mESCs showed the presence of increased numbers of lagging chromosomes and anaphase bridges compared with WT control cells (Figure 4C).

In addition to promoting faithful chromosome segregation, cohesion is important for mediating recombination in response to DNA damage, and loss of cohesion leads to structural CIN (Xu et al., 2011). Consequently, mammalian cells with defects in cohesion are hypersensitive to a number of DNA-damaging agents, including the crosslinking agent mitomycin C (MMC) (van der Lelij et al., 2009; Vrouwe et al., 2007). We therefore tested whether loss of BAF180 would sensitize cells to MMC. We found that both BAF180$^{-/-}$ mESCs and BAF180-depleted 1BR-hTERT human cells were more sensitive to MMC than the respective WT cells (Figure 5A). These data suggest that loss of cohesion in the absence of BAF180 leads to a deficiency in recombination-based repair of DNA damage, leading to decreased viability after MMC exposure.

This defect would be expected to further exacerbate CIN in the absence of BAF180 under these conditions. Consistent with this idea, we found that the BAF180$^{-/-}$ mESCs had a clear increase in structural aberrations and greater incidence of micronuclei than the WT controls following exposure to MMC (Figures 5B and 5C; Table 1). Strikingly, we also found that MMC treatment led to a profound effect on aneuploidy in the BAF180$^{-/-}$ cells (Figure 5D), providing direct evidence of dynamic CIN in these cells. We also found that the shBAF180 U2OS cells, where p21 transcripts are not decreased relative to the shControl cells, also showed evidence of MMC-induced genome instability (Figure S5). Collectively, these data demonstrate that BAF180 plays a key role in preventing CIN.

DISCUSSION

We found that BAF180 is important for the establishment or maintenance of cohesion on chromatin at centromeres. Interestingly, BAF180 is enriched at kinetochores of chromosomes during mitosis (Xue et al., 2000), and there is evidence from budding yeast that kinetochores promote cohesin loading by Scc2-Scc4 (Natsume et al., 2013), raising the possibility that PBAF works with kinetochores in some way to promote this function.

We found that cancer-associated mutations in BAF180 are compromised for cohesion and cohesion-dependent functions

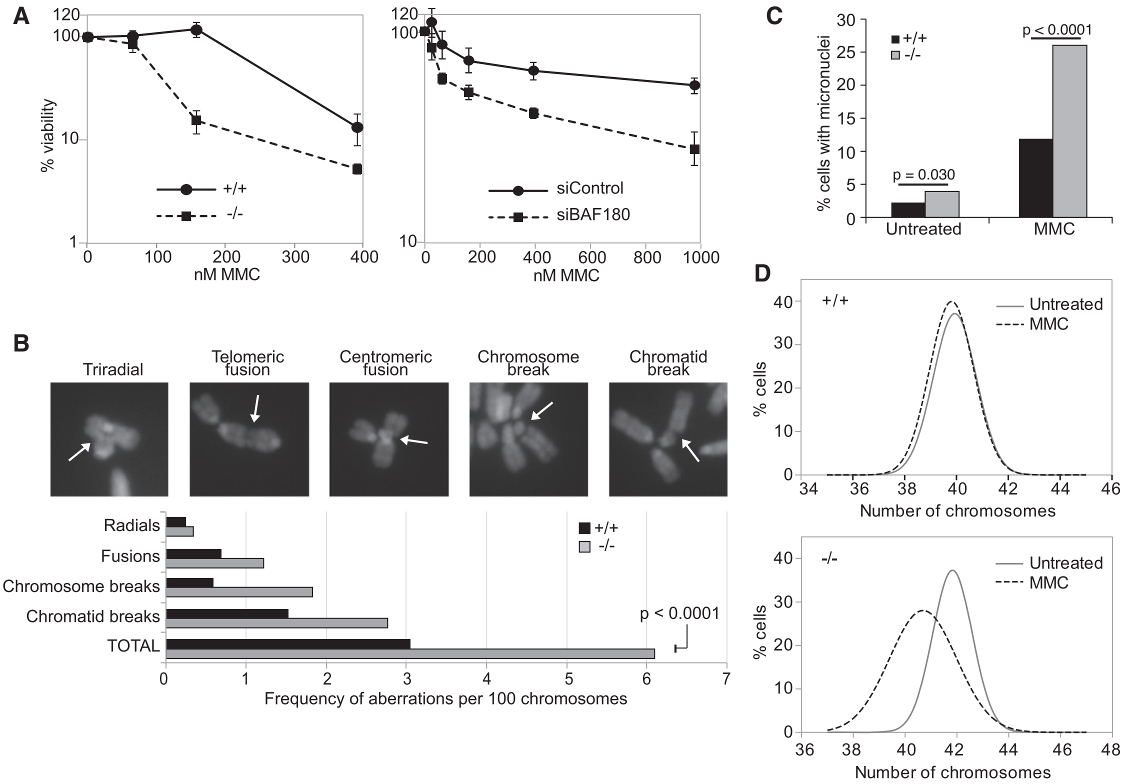

Figure 5. Loss of BAF180 Leads to Hypersensitivity to DNA Damage, Increased Frequency of Chromosome Aberrations, and Dynamic CIN

(A) Viability curves of WT (+/+) or BAF180 knockout (−/−) mESCs (left panel), and control and BAF180-depleted 1BR-hTERT cells (right panel) following exposure to MMC.

(B) The presence of chromosomal aberrations in metaphase spreads prepared from WT (+/+) or BAF180 knockout (−/−) mESCs following exposure to MMC was analyzed and presented as frequency per 100 chromosomes. Images representative of each category are shown in the top panels.

(C) Quantification of micronuclei present in WT (+/+) or BAF180 knockout (−/−) mESCs following exposure to MMC.

(D) Distribution curves showing chromosome numbers from WT (+/+) and BAF180 knockout (−/−) mESCs following exposure to MMC.

See also Figure S5.

in both yeast and mammalian cells, demonstrating profound conservation of function. These findings, together with the CIN that we find in the absence of BAF180, support the notion that the ability of BAF180 to promote cohesion is important for preventing tumorigenesis. Notably, these data are not incompatible with the idea that BAF180 also prevents tumorigenesis via its role in regulating transcription; rather, they uncover an additional mechanism. In fact, the ability of BAF180 (and PBAF) to function in multiple pathways to prevent tumorigenesis may make it particularly critical as a tumor-suppressor gene, and may explain the frequency with which subunits of the complex are found mutated in cancer samples.

We find evidence of both structural and numerical CIN. Loss of centromeric cohesion is likely to disrupt accurate chromosome segregation and would therefore lead to numerical CIN. Although it is less obvious how a defect in cohesion at the centromere would lead to structural CIN, evidence of both structural and numerical CIN has been reported for cells lacking other members of the centromere-specific cohesion pathway, i.e., SA2 and PDS5B (Brough et al., 2012; Carretero et al., 2013; Kong et al., 2014; Solomon et al., 2011). Cohesin is recruited to sites of DNA damage in order to promote recombination-based repair, and in yeast this is dependent on RSC (Oum et al., 2011). Interestingly, a recent report suggests that SA2, but not SA1, is recruited to sites of DNA damage (Kong et al., 2014). Together, these findings raise the intriguing possibility that the establishment of cohesion in response to DNA damage is mediated specifically by the centromere-specific cohesion pathway.

These results demonstrate a role for BAF180 in promoting genome stability that is distinct from its well-characterized role in transcriptional regulation. The discovery that BAF180 contributes to cohesion suggests potential directions for therapeutic interventions for BAF180-deficient tumors.

EXPERIMENTAL PROCEDURES

Cell Culture Conditions, Strains, Plasmids, and Antibodies

For details regarding the cell culture conditions, strains, plasmids, and antibodies used in this work, see Supplemental Experimental Procedures.

Metaphase Spreads

To arrest cells in metaphase, the cells were treated with 0.1 μg/ml colcemid for 3 hr, trypsinized, swollen in 75 mM KCl for 20 min at room temperature, and fixed in Carnoy's fixative (methanol/acetic acid 3:1). Cells were spotted onto a slide floating in a 37°C waterbath and dried overnight at room temperature.

Table 1. Average Number and Range per Cell of Chromosomal Aberrations in mESCs following MMC Exposure

Category	+/+	−/−
Chromatid breaks	0.5 (0–3)	2.2 (0–14)
Chromosome breaks	0.3 (0–4)	0.8 (0–10)
Triradial	0.1 (0–2)	0.1 (0–1)
Quadriradial	0 (0–0)	0.02 (0–1)
Fusion	0.3 (0–2)	0.5 (0–8)
Total	2.2 (0–6)	4.6 (0–32)

DNA staining was performed using ProLong Gold Antifade Reagent (Life Technologies) with DAPI. For the mESC cohesion assays, 200 spreads were analyzed for each genotype. Centromeric cohesion was scored as "normal" when fewer than three chromosomes showed gaps between sister chromatid centromeres, or "defective" when more than two chromosomes showed gaps. Arm cohesion was scored as "normal" when fewer than three chromosomes showed fully separated chromosome arms, or "defective" when more than two chromosomes showed fully separated chromosome arms. For chromosome counts, 100 cells were analyzed for each cell type, with and without treatment with 0.04 μg/ml MMC for 40 hr prior to metaphase arrest. The same conditions were used for analysis of structural chromosome aberrations, with 2,033 and 2,133 chromosomes analyzed for +/+ and −/− mESCs, respectively.

FISH

1BR-hTERT cells transfected with siControl or siBAF180 were treated with 0.1 μg/ml colcemid for 3 hr to arrest in metaphase before mitotic cells were dislodged. Cells were washed with PBS and fixed in Carnoy's fixative. FISH was performed essentially as described previously (Canudas and Smith, 2009). Cells were hybridized overnight with either DNA probes against arm and centromere regions of chromosome 10 (DiGeorge II probe, LPU015; Cytocell) or subtelomeric regions of chromosome 16 (chromosome 16ptel05 probe, LPT16R; Cytocell) according to the manufacturer's protocol. Nuclei were stained with ProLong Gold Antifade Reagent with DAPI.

Transcript Analysis

RNA was extracted from the indicated cell lines using an RNeasy kit (QIAGEN). Then 1 μg of RNA was reverse transcribed into cDNA for analysis by qPCR using primers specific to the indicated locus (using QuantiTect Primer Mix from QIAGEN).

IF-FISH

For IF-FISH, 4×10^5 U20S cells were plated onto glass coverslips and transfected with 20 nM BAF180 single siRNA (Invitrogen) or nontargeting control siRNA using HiPerFect transfection reagent (QIAGEN). After 19 hr, cells were treated with 2.5 mM thymidine for 17 hr before being released. At 9 hr after release, cells were transfected with the siRNA-resistant plasmids pEGFP, pEGFP-BAF180r, pEGFP-BAF180r-T232P, and pEGFP-BAF180r-M538I using NanoJuice Core transfection reagent (Merck Chemicals). After 5 hr, the cells were washed with PBS and subjected to a second thymidine block using 2.5 mM thymidine for a further 17 hr. At 8 hr after release, the cells were fixed with 3% paraformaldehyde (PFA).

For IF, cells were blocked in blocking solution (3% BSA, 0.1% Triton X-100, 1 mM EDTA pH 8.0 in PBS) for 30 min at room temperature, and subjected to antibody incubation in blocking solution. Immunostained cells were fixed in 3% PFA for 10 min at room temperature. DNA FISH was performed according to Chaumeil et al. (2008). Nuclei were stained with ProLong Gold Antifade Reagent with DAPI.

Yeast Survival and Recombination Assays

Cultures of Δ*rsc2* DMY3010 strain transformed with pRsc2-myc, pRsc2-T67P-myc, pRsc2-M280I-myc, pRsc2-H458P-myc, or pRsc2-D540G-myc plasmids were grown to mid-log phase in synthetic complete media lacking tryptophan. Serial dilutions were spotted or plated onto plates containing the indicated drug. Colonies for survival assays were counted 3 days after plating. Recombination assays were performed as described previously (Huang et al., 2006). Ten- to twenty-thousand colonies were analyzed per strain.

Analysis of Micronuclei and Aberrant Mitoses

For mESCs, spontaneous micronuclei were counted in interphase cells from two independent experiments (total counts: 983 +/+ cells, 1,043 −/− cells). For 1BR-hTERT cells, three separate knockdown experiments were analyzed (total counts: 2,629 for siControl, 2,716 for siBAF180). In experiments to analyze the effect of MMC on micronuclei formation, 563 +/+ and 676 −/− cells were counted. DNA staining was performed using ProLong Gold Antifade Reagent with DAPI. Analysis of aberrant mitoses was performed on 183 +/+ and 207 −/− anaphase mESCs from two independent experiments.

Viability Assays

Viability assays following treatment with MMC were performed in 96-well format in triplicate at the stated doses; 1×10^4 mESCs and 5×10^3 1BR-hTERT cells were plated per well. Viability was analyzed 4 days following treatment using CellTiter-Glo Reagent (Promega).

Statistical Analyses

Frequency counts for cohesion scoring, spontaneous micronuclei, abnormal anaphases, and structural chromosome aberration assays were analyzed with a two-tailed Fisher's exact test using GraphPad software. Distribution counts from FISH experiments were analyzed using the Kolmogorov-Smirnov test. Differences in recombination frequency and survival assays were analyzed with an unpaired two-tailed t test using GraphPad software. Significance was indicated as *$p < 0.05$, **$p < 0.01$, ***$p < 0.001$.

SUPPLEMENTAL INFORMATION

Supplemental Information includes Supplemental Experimental Procedures and five figures and can be found with this article online at http://dx.doi.org/10.1016/j.celrep.2014.02.012.

ACKNOWLEDGMENTS

We thank Danesh Moazed for yeast strain DMY3010, Zhong Wang for the BAF180 knockout mESCs, Suzanna Hopkins and Andreas Kakarougkas for help generating cell lines and reagents, and Penny Jeggo and members of the Downs lab for helpful discussions. P.M.B., A.L.C., and J.A.D. are supported by Cancer Research UK CEA C7905/A10269. R.C. and A.B. acknowledge support from the Medical Research Council (grant G0701428 to A.B.).

Received: September 3, 2013
Revised: January 28, 2014
Accepted: February 10, 2014
Published: March 6, 2014

REFERENCES

Angus-Hill, M.L., Schlichter, A., Roberts, D., Erdjument-Bromage, H., Tempst, P., and Cairns, B.R. (2001). A Rsc3/Rsc30 zinc cluster dimer reveals novel roles for the chromatin remodeler RSC in gene expression and cell cycle control. Mol. Cell *7*, 741–751.

Baetz, K.K., Krogan, N.J., Emili, A., Greenblatt, J., and Hieter, P. (2004). The ctf13-30/CTF13 genomic haploinsufficiency modifier screen identifies the yeast chromatin remodeling complex RSC, which is required for the establishment of sister chromatid cohesion. Mol. Cell. Biol. *24*, 1232–1244.

Barber, T.D., McManus, K., Yuen, K.W., Reis, M., Parmigiani, G., Shen, D., Barrett, I., Nouhi, Y., Spencer, F., Markowitz, S., et al. (2008). Chromatid cohesion defects may underlie chromosome instability in human colorectal cancers. Proc. Natl. Acad. Sci. USA *105*, 3443–3448.

Brough, R., Bajrami, I., Vatcheva, R., Natrajan, R., Reis-Filho, J.S., Lord, C.J., and Ashworth, A. (2012). APRIN is a cell cycle specific BRCA2-interacting

protein required for genome integrity and a predictor of outcome after chemotherapy in breast cancer. EMBO J. *31*, 1160–1176.

Brownlee, P.M., Chambers, A.L., Oliver, A.W., and Downs, J.A. (2012). Cancer and the bromodomains of BAF180. Biochem. Soc. Trans. *40*, 364–369.

Burrows, A.E., Smogorzewska, A., and Elledge, S.J. (2010). Polybromo-associated BRG1-associated factor components BRD7 and BAF180 are critical regulators of p53 required for induction of replicative senescence. Proc. Natl. Acad. Sci. USA *107*, 14280–14285.

Canudas, S., and Smith, S. (2009). Differential regulation of telomere and centromere cohesion by the Scc3 homologues SA1 and SA2, respectively, in human cells. J. Cell Biol. *187*, 165–173.

Carretero, M., Ruiz-Torres, M., Rodríguez-Corsino, M., Barthelemy, I., and Losada, A. (2013). Pds5B is required for cohesion establishment and Aurora B accumulation at centromeres. EMBO J. *32*, 2938–2949.

Chambers, A.L., Brownlee, P.M., Durley, S.C., Beacham, T., Kent, N.A., and Downs, J.A. (2012). The two different isoforms of the RSC chromatin remodeling complex play distinct roles in DNA damage responses. PLoS ONE *7*, e32016.

Chaumeil, J., Augui, S., Chow, J.C., and Heard, E. (2008). Combined immunofluorescence, RNA fluorescent in situ hybridization, and DNA fluorescent in situ hybridization to study chromatin changes, transcriptional activity, nuclear organization, and X-chromosome inactivation. Methods Mol. Biol. *463*, 297–308.

Guo, G., Sun, X., Chen, C., Wu, S., Huang, P., Li, Z., Dean, M., Huang, Y., Jia, W., Zhou, Q., et al. (2013). Whole-genome and whole-exome sequencing of bladder cancer identifies frequent alterations in genes involved in sister chromatid cohesion and segregation. Nat. Genet. *45*, 1459–1463.

Holland, A.J., and Cleveland, D.W. (2012). Losing balance: the origin and impact of aneuploidy in cancer. EMBO Rep. *13*, 501–514.

Huang, J., and Moazed, D. (2003). Association of the RENT complex with nontranscribed and coding regions of rDNA and a regional requirement for the replication fork block protein Fob1 in rDNA silencing. Genes Dev. *17*, 2162–2176.

Huang, J., Hsu, J.M., and Laurent, B.C. (2004). The RSC nucleosome-remodeling complex is required for Cohesin's association with chromosome arms. Mol. Cell *13*, 739–750.

Huang, J., Brito, I.L., Villén, J., Gygi, S.P., Amon, A., and Moazed, D. (2006). Inhibition of homologous recombination by a cohesin-associated clamp complex recruited to the rDNA recombination enhancer. Genes Dev. *20*, 2887–2901.

Kong, X., Ball, A.R., Jr., Pham, H.X., Zeng, W., Chen, H.Y., Schmiesing, J.A., Kim, J.S., Berns, M., and Yokomori, K. (2014). Distinct functions of human cohesin-SA1 and cohesin-SA2 in DSB repair. Mol. Cell. Biol. *34*, 685–698.

Musio, A., Montagna, C., Zambroni, D., Indino, E., Barbieri, O., Citti, L., Villa, A., Ried, T., and Vezzoni, P. (2003). Inhibition of BUB1 results in genomic instability and anchorage-independent growth of normal human fibroblasts. Cancer Res. *63*, 2855–2863.

Natsume, T., Müller, C.A., Katou, Y., Retkute, R., Gierliński, M., Araki, H., Blow, J.J., Shirahige, K., Nieduszynski, C.A., and Tanaka, T.U. (2013). Kinetochores coordinate pericentromeric cohesion and early DNA replication by Cdc7-Dbf4 kinase recruitment. Mol. Cell *50*, 661–674.

Oum, J.H., Seong, C., Kwon, Y., Ji, J.H., Sid, A., Ramakrishnan, S., Ira, G., Malkova, A., Sung, P., Lee, S.E., and Shim, E.Y. (2011). RSC facilitates Rad59-dependent homologous recombination between sister chromatids by promoting cohesin loading at DNA double-strand breaks. Mol. Cell. Biol. *31*, 3924–3937.

Remeseiro, S., Cuadrado, A., Carretero, M., Martínez, P., Drosopoulos, W.C., Cañamero, M., Schildkraut, C.L., Blasco, M.A., and Losada, A. (2012). Cohesin-SA1 deficiency drives aneuploidy and tumourigenesis in mice due to impaired replication of telomeres. EMBO J. *31*, 2076–2089.

Shain, A.H., and Pollack, J.R. (2013). The spectrum of SWI/SNF mutations, ubiquitous in human cancers. PLoS ONE *8*, e55119.

Solomon, D.A., Kim, T., Diaz-Martinez, L.A., Fair, J., Elkahloun, A.G., Harris, B.T., Toretsky, J.A., Rosenberg, S.A., Shukla, N., Ladanyi, M., et al. (2011). Mutational inactivation of STAG2 causes aneuploidy in human cancer. Science *333*, 1039–1043.

Solomon, D.A., Kim, J.S., Bondaruk, J., Shariat, S.F., Wang, Z.F., Elkahloun, A.G., Ozawa, T., Gerard, J., Zhuang, D., Zhang, S., et al. (2013). Frequent truncating mutations of STAG2 in bladder cancer. Nat. Genet. *45*, 1428–1430.

van der Lelij, P., Godthelp, B.C., van Zon, W., van Gosliga, D., Oostra, A.B., Steltenpool, J., de Groot, J., Scheper, R.J., Wolthuis, R.M., Waisfisz, Q., et al. (2009). The cellular phenotype of Roberts syndrome fibroblasts as revealed by ectopic expression of ESCO2. PLoS ONE *4*, e6936.

Varela, I., Tarpey, P., Raine, K., Huang, D., Ong, C.K., Stephens, P., Davies, H., Jones, D., Lin, M.L., Teague, J., et al. (2011). Exome sequencing identifies frequent mutation of the SWI/SNF complex gene PBRM1 in renal carcinoma. Nature *469*, 539–542.

Vrouwe, M.G., Elghalbzouri-Maghrani, E., Meijers, M., Schouten, P., Godthelp, B.C., Bhuiyan, Z.A., Redeker, E.J., Mannens, M.M., Mullenders, L.H., Pastink, A., and Darroudi, F. (2007). Increased DNA damage sensitivity of Cornelia de Lange syndrome cells: evidence for impaired recombinational repair. Hum. Mol. Genet. *16*, 1478–1487.

Wang, Z., Zhai, W., Richardson, J.A., Olson, E.N., Meneses, J.J., Firpo, M.T., Kang, C., Skarnes, W.C., and Tjian, R. (2004). Polybromo protein BAF180 functions in mammalian cardiac chamber maturation. Genes Dev. *18*, 3106–3116.

Xia, W., Nagase, S., Montia, A.G., Kalachikov, S.M., Keniry, M., Su, T., Memeo, L., Hibshoosh, H., and Parsons, R. (2008). BAF180 is a critical regulator of p21 induction and a tumor suppressor mutated in breast cancer. Cancer Res. *68*, 1667–1674.

Xu, H., Tomaszewski, J.M., and McKay, M.J. (2011). Can corruption of chromosome cohesion create a conduit to cancer? Nat. Rev. Cancer *11*, 199–210.

Xue, Y., Canman, J.C., Lee, C.S., Nie, Z., Yang, D., Moreno, G.T., Young, M.K., Salmon, E.D., and Wang, W. (2000). The human SWI/SNF-B chromatin-remodeling complex is related to yeast rsc and localizes at kinetochores of mitotic chromosomes. Proc. Natl. Acad. Sci. USA *97*, 13015–13020.

SUPPLEMENTAL INFORMATION

BAF180 promotes cohesion and prevents genome instability and aneuploidy

Brownlee, *et al.*

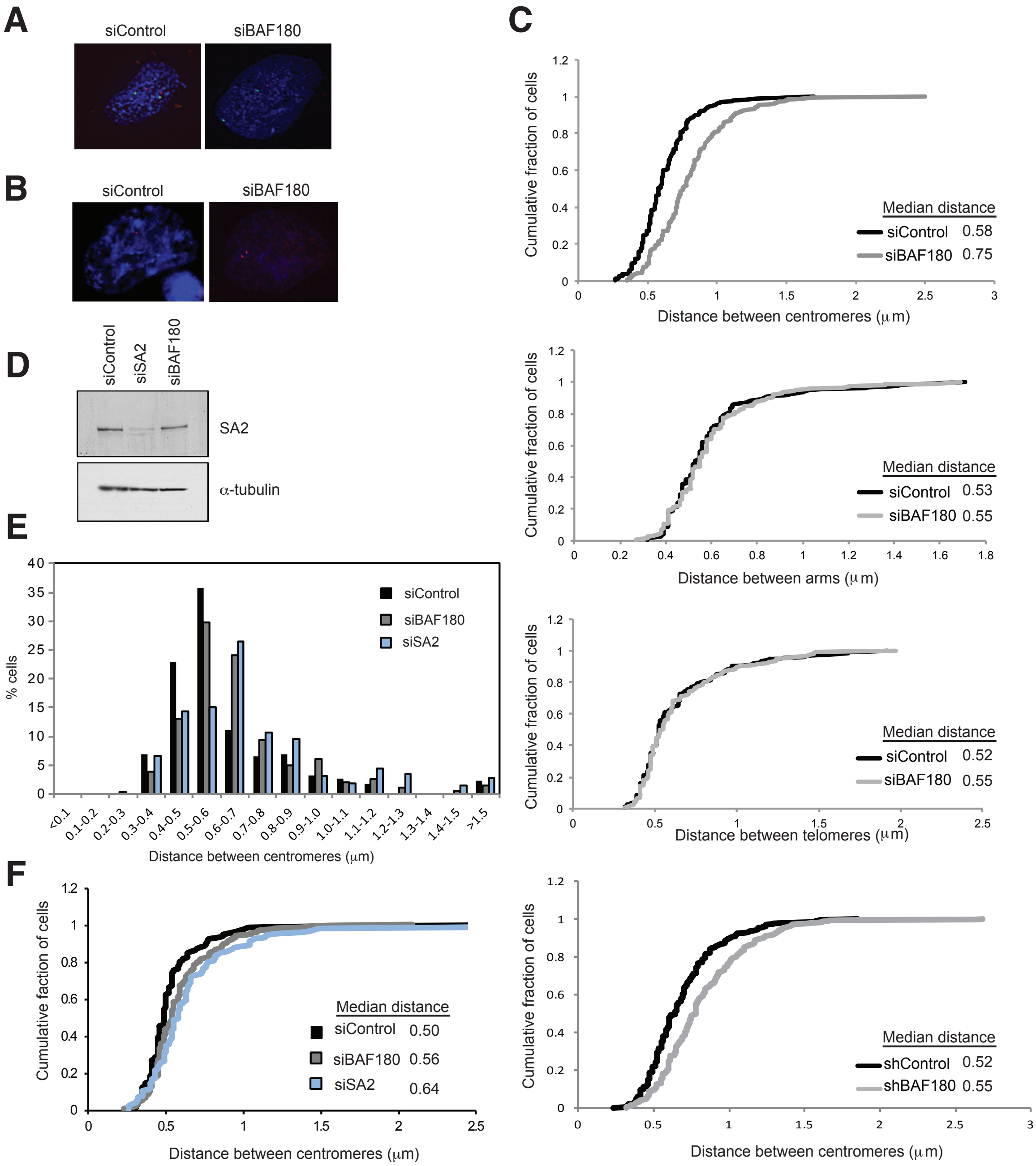

Fig. S1. Depletion of BAF180 results in defective cohesion at centromeres, Related to Figure 1. (A) Representative images of FISH analyses of si Control and siBAF180 1BR hTERT cells using a mixture of probes directed against the centromere (green) or arm (red) of chromosome 10. (B) Representative images of FISH analyses of siControl and siBAF180 1BR hTERT cells using a probe directed against the subtelomeric region of chromosome 16. (C) The data generated using these probes, presented in Figures 1D and F, are replotted here as cumulative plots and the median distances between sister chromatids is shown in each panel. Top three panels are of 1BR hTERT cells and the bottom panel is of the stable U2OS cell lines. (D) Western blot analysis of siRNA treated 1BR hTERT cells using antibodies directed against SA2 or tubulin. (E and F) siRNA treated cells as in D were analysed by FISH and distances between centromeres were measured and plotted as a distribution (E) or as a cumulative plot (F). Median distances are indicated in (F) and both BAF180- and SA2-depleted cells show a significant difference in distribution from control cells ($p=0.001$ and $p<0.001$, respectively).

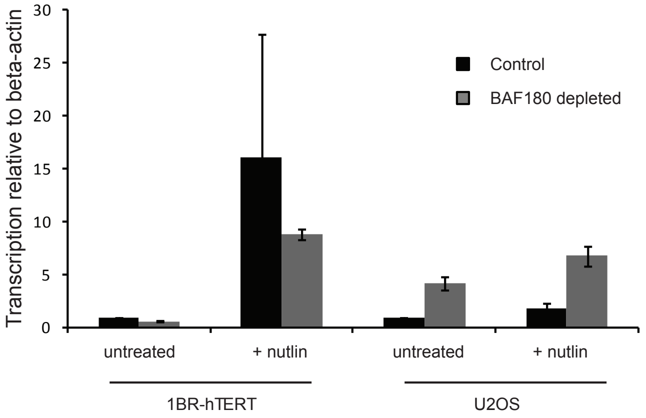

Fig S2. p21 transcription in 1BR-hTert and U20S cell lines in the absence and presence of nutlin, Related to Figure 2. Quantitative RT-PCR analysis of p21 transcripts in siControl and siBAF180 1BR hTERT cells (left) and shControl and shBAF180 U2OS cells (right) in the absence and presence of nutlin. Data are the average of three independent experiments and are expressed relative to a control transcript; beta-actin. For each cell line, the data have been normalized to the untreated control.

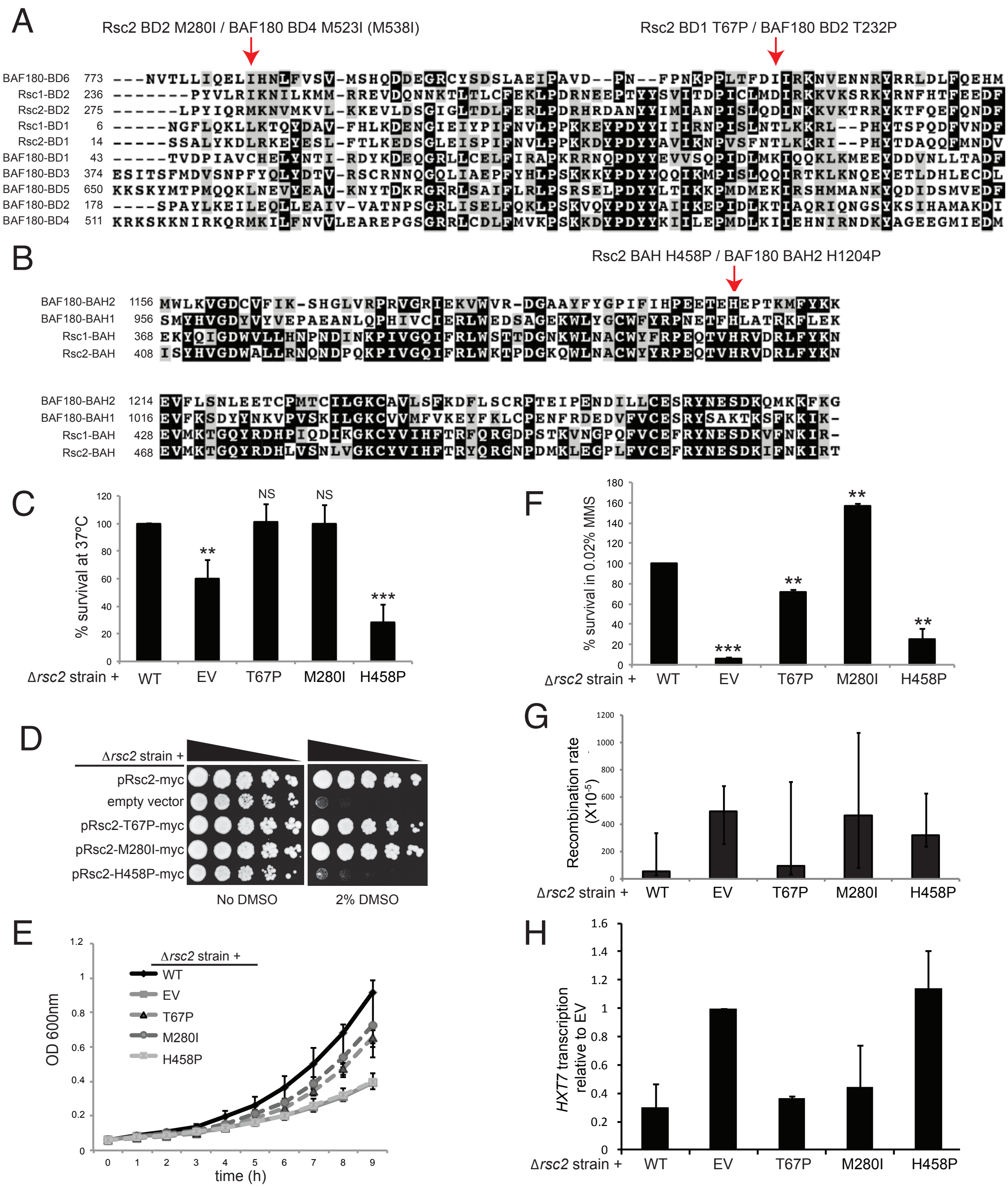

Fig. S3. Cancer associated mutations of BAF180 are conserved in the yeast homologues, and mutation impairs a subset of Rsc2 functions, Related to Figure 3. **(A)** Pilup of all six bromodomains of BAF180 with the bromodomains of Rsc1 and Rsc2. The cancer associated mutations of BAF180 are indicated (M523 corresponds to M538 of BAF180 isoform 8, used in our assays). **(B)** Pileup of the two BAH domains of BAF180 with the BAH domains from Rsc1 and Rsc2. The cancer associated H-P mutation is indicated. **(C)** Quantification of survival at 37ºC (ts phenotype) of wt and Rsc2 mutants. **(D)** Serial dilutions of mid-log cultures of the indicated strains were plated onto media with and without DMSO. **(E)** Growth curves of wt and mutant Rsc2-containing strains. **(F)** Quantification of survival in the presence of the DNA damaging agent methyl methane sulfonate (MMS) of wt and Rsc2 mutants. **(G)** Median rates of recombination with 10% and 90% confidence intervals (bars). **(H)** Quantitative RT-PCR analysis in *rsc2* cells harboring the indicated plasmid.

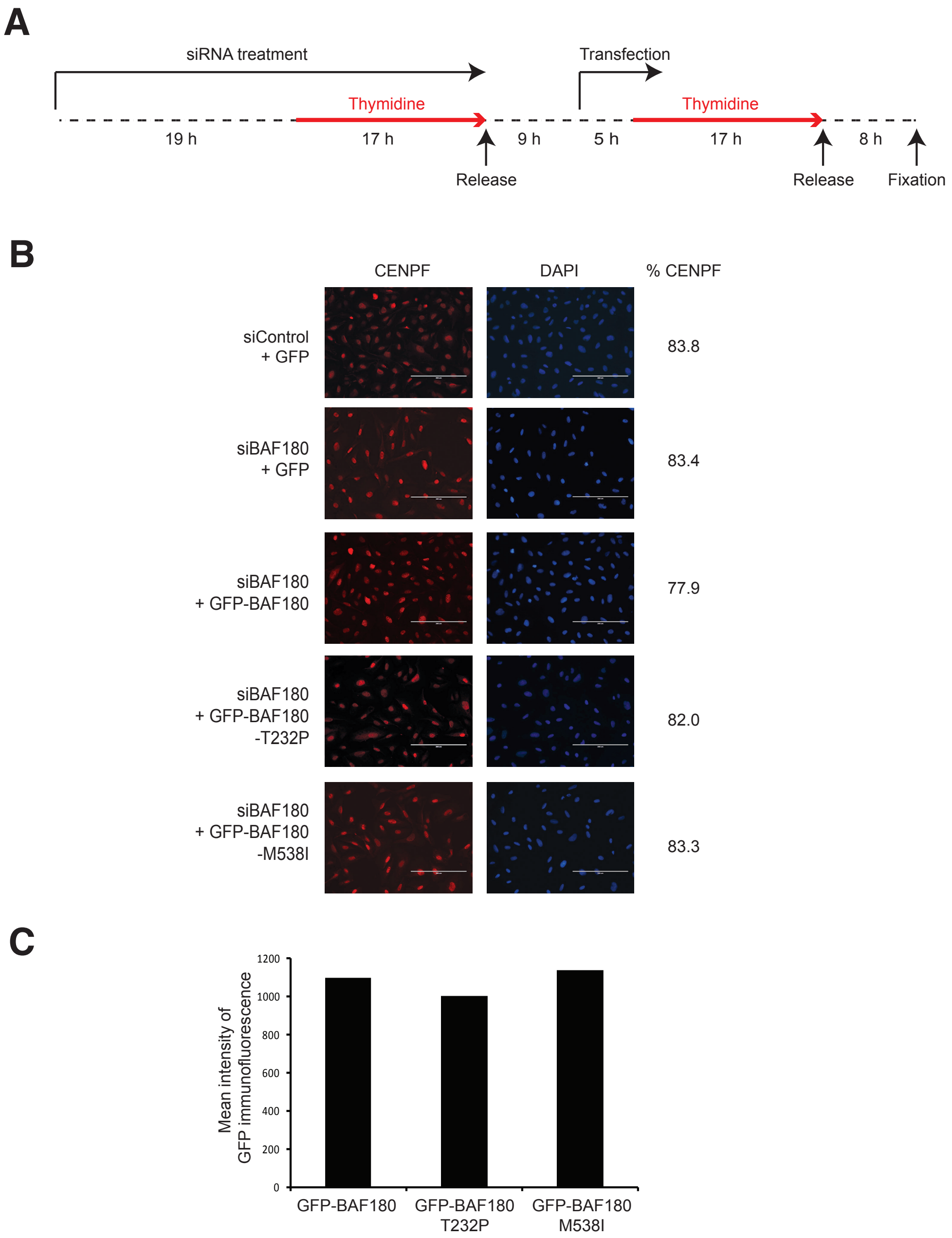

Fig. S4. Preparation of cells for cohesin analysis as in Figure 3. **(A)** Illustration of the protocol used to deplete BAF180, transfect with GFP constructs as in (B) and synchronize the cells in G2. **(B)** Representative images showing CENPF immunostaining, used as a marker of G2 cells. The percentage of CENPF positive cells is shown on the right. **(C)** Analysis of expression of GFP-BAF180 and mutants by immunofluorescence. Slides were prepared as in (A) and mean intensities were calculated from analysis of at least 60 cells.

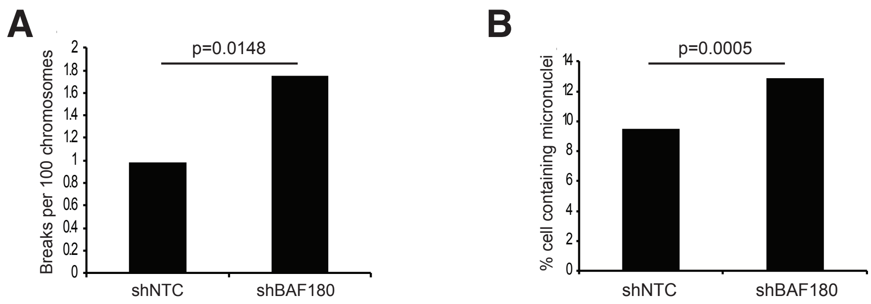

Fig. S5. Loss of BAF180 in U2OS cells leads to increased frequency of breaks and chromosomal instability on exposure to MMC, Related to Figure 5. **(A)** Analysis of chromosome and chromatid breaks in control or BAF180-depleted U2OS cells following exposure to 0.04 μg/ml MMC for 48 hours. A total of 2862 or 2741 chromosomes were analysed for control and BAF180-depleted cells, respectively, and data are expressed as breaks per 100 chromosomes. (B) Quantification of micronuclei in control or BAF180-depleted U2OS cells following exposure to MMC as in (A). A minimum of 1500 cells was scored for each cell line.

EXTENDED EXPERIMENTAL PROCEDURES

Cell culture

Mouse ES cells were cultured at 37°C in a 5% CO_2 incubator in Knockout™ DMEM (Gibco), supplemented with 15% ES-Cult FBS (Stemcell Technologies), 1% penicillin/streptomycin, 1% NEAA, 1% L-glutamine, 0.1 μM β-mercaptoethanol and 1000 U/ml LIF (Millipore). Cells were seeded into gelatin-coated dishes. 1BR-hTERT and U2OS cells were cultured at 37°C in a 5% CO_2 incubator in DMEM (Gibco) supplemented with 15% FBS, 1% penicillin/streptomycin and 1% L-glutamine.

siRNA knock-downs

1BR-hTERT and U2OS cells were plated at $1x10^5$ cells per 6cm dish and knock-downs performed as (Niimi et al., 2012). Cells were transfected with 20 nM BAF180 siRNA or 20 nM non-targeting control (ON-TARGET plus SMART pool, Dharmacon) using HiPerFect transfection reagent (Qiagen), and then again with the same siRNAs 24h later. Cells were treated or sampled 72h after the first transfection.

Antibodies and immunoblotting

Custom made and commercially available antibodies used in this study are as follows: Myc (9E10 clone, CRUK), BAF180 (A301-590A, Universal Biologicals Cambridge), Smc1 (A300-055A, Universal Biologicals Cambridge), Smc3 (ab155587, Abcam), SA1 (ab4457, Abcam), SA2 (ab4463, Abcam), β-actin (ab8226, Abcam), H3 (Abcam), α-tubulin (ab7750, Abcam), CENPF (ab108483, Abcam), GFP (A6455, Life Technologies), H2A (Downs et al., 2000). Samples for Western blot analysis were prepared from yeast by glass-bead disruption and TCA precipitation. For mammalian cell samples, cells were lysed by resuspension in Laemmli buffer.

Yeast strains and plasmids

RSC2 was deleted in DMY3010 (Huang et al., 2006) or BY471 backgrounds via standard gene-disruption methods using a cassette amplified by PCR. pRsc2-myc

(Chambers et al., 2013) was altered by site-directed mutagenesis to generate pRsc2-T67P-myc, pRsc2-M280I-myc, pRsc2-H458P-myc and pRsc2-D540G.

BAF180 expression plasmids

BAF180 complete cDNA (clone MGC:156155, IMAGE:40082629) was purchased from Source BioScience and cloned into the *Hind*III/*Kpn*I sites of pEGFP-C3 (Clontech) to generate pEGFP-BAF180. This clone contains a N122S substitution relative to the published sequence. To create the siRNA resistant construct pEGFP-BAF180r site-directed mutagenesis was performed using the following oligonucleotide sequence: GAGAAATCTTGAGACAGCCAAGAAA. Site-directed mutagenesis was performed on pEGFP-BAF180r to generate siRNA resistant plasmids expressing EGFP-tagged BAF180 containing substitutions identified in tumour samples: pEGFP-BAF180r-T232P and pEGFP-BAF180r-M538I.

Analysis of yeast transcription

Δ*rsc2* DMY3010 containing pRS415 (empty vector), pRsc2-myc (wt), pRsc2-T67P-myc, pRsc2-M280I-myc or pRsc2-H458P-myc were grown to mid-log phase in media lacking tryptophan and RNA extracted using an RNeasy kit (Qiagen). 1 μg of RNA was reverse transcribed into cDNA for analysis by qPCR using primers specific to the *HXT7* locus.

SUPPLEMENTAL REFERENCES

Chambers, A.L., Pearl, L.H., Oliver, A.W., and Downs, J.A. (2013). The BAH domain of Rsc2 is a histone H3 binding domain. Nucleic Acids Res *41*, 9168-9182.

Downs, J.A., Lowndes, N.F., and Jackson, S.P. (2000). A role for Saccharomyces cerevisiae histone H2A in DNA repair. Nature *408*, 1001-1004.

Huang, J., Brito, I.L., Villen, J., Gygi, S.P., Amon, A., and Moazed, D. (2006). Inhibition of homologous recombination by a cohesin-associated clamp complex recruited to the rDNA recombination enhancer. Genes Dev *20*, 2887-2901.

Niimi, A., Chambers, A.L., Downs, J.A., and Lehmann, A.R. (2012). A role for chromatin remodellers in replication of damaged DNA. Nucleic acids research.
